# Suicide Behaviors in Adult Inpatients with Mental Disorders in Beijing, China

**DOI:** 10.3390/ijerph14030259

**Published:** 2017-03-03

**Authors:** Qi Gao, Hua Fan, Fei Di, Xue Xia, Haiying Long, Huiping Zhu

**Affiliations:** 1 School of Public Health, Capital Medical University, 10 Xitoutiao, Youanmenwai, Beijing 100069, China; gaoqi@ccmu.edu.cn (Q.G.); xiax0708@163.com (X.X.); 2 Beijing Municipal Key Laboratory of Clinical Epidemiology, Beijing 100069, China; 3 Department of Psychiatry, Beijing Anding Hospital, Capital Medical University, Beijing 100088, China; iy4232925@163.com; 4 Department of Neurosurgery, Beijing Tian Tan Hospital, Capital Medical University, Beijing 100050, China; difeittyy@aliyun.com

**Keywords:** suicide, Chinese subjects, psychiatry

## Abstract

*Background*: This study examined the tendency and suicidal behavior rates of Chinese adult inpatients with different types of mental disorders from 2010 to 2015. The aim was to provide some interesting clues for further studies. *Methods*: Adult patients with mental disorders who were hospitalized in Beijing Anding hospital from 1 January 2010 to 31 December 2015 were included. Chi-square tests were used to compare the difference among inpatients with mental disorders by gender and year. Frequency, trend and suicidal behavior rates of inpatients with mental disorders were graphed. *Results*: A total of 17,244 psychiatric adult inpatients were included in our study. About 53.2% of the inpatients had mood disorders, followed by schizophrenia, which accounted for 34.6%. The proportion of female inpatients with mental disorders was larger than that of males (52.6% to 47.4%). Of the total, 3296 psychiatric inpatients were recognized as having suicidal behaviors. The rate of suicidal behavior among all inpatients was 19.1%, and it varied over the years. The suicidal behavior rate of female inpatients with mood disorders was much higher than that of the corresponding male inpatients. *Conclusions*: The presence of suicidal behavior varied among people with different types of mental disorders. For each type of mental illness, identifying the risk of specific suicide behavior would help tailor-make preventive efforts accordingly.

## 1. Introduction

Suicide is among the leading causes of death and injury around the world, with over 800,000 people dying or one death every 40 s by suicide every year [1,2]. In China, suicide is the fifth most important cause of death, with around 287,000 suicide deaths per year during 1995 to 1999, accounting for up to a third of suicides worldwide [3,4]. It is estimated that 48~500 million people are thought to experience suicide bereavement each year, which leads to a series of social and public health issues [1]. Thus, people who experience such a loss, the wider public and health professionals often struggle to understand this complicated behavior, although in many cultures the topic still remains a taboo. 

Mental health is a fundamental and inseparable component of the WHO’s definition of health [1]. Most notably, mental disorders contribute to mortality through suicide [5,6]. They represent the most commonly studied risk factor for suicide, and in high-income countries the link between mental disorders and suicide is well established [1,7,8,9,10,11]. 

Although studies from western countries showed over 90% of suicides were associated with mental disorders [11,12], in China, where about 60% of suicide victims had a psychiatric diagnosis, this proportion was substantially lower [13]. In China, it was estimated that 173 million adults had a diagnosable psychiatric disorder, however, mental health services have been a low priority [8,14]. Suicide prevention as well as strategies to tackle stigma and discrimination against people with mental disorders were absent from the China’s national work plan for mental health 2015–2020 [9].

At present, the degree of psychopathology underpinning suicide cannot be clearly evaluated. For suicide prevention, there is still a lot of work remaining to be done for people with mental disorders. It is still unknown whether the suicide rate and risk differed in each category of mental disorders in China. In this study, we examined the admission tendencies of inpatients with mental disorder and tendency of the suicidal behavior rates during recent years. We conducted a preliminary examination of the association between suicidal behaviors and some common mental disorders that were generally regarded with a high level of concern by using inpatient data from the Beijing Anding Hospital (Beijing, China). 

## 2. Methods

### 2.1. Data Collection

Patients with mental disorders who were hospitalized in Beijing Anding hospital—one of the top five psychiatric hospitals in China—were consecutively collected from 1 January 2010 to 31 December 2015. The Beijing Anding Hospital receives psychiatric patients mainly from the Beijing area. There were 800 hospital beds in Beijing Anding Hospital, which was unchanged from 2010 to 2015. The trend of inpatient numbers in this hospital would highly reflect the trend of numbers of psychiatric patients in Beijing during the 6 years. All of the medical records of the inpatients were collected for this study. Our study was conducted in accordance with the Declaration of Helsinki, and was reviewed and approved by the Institutional Review Board of Capital Medical University (2014SY37).

The inclusion criteria were: (a) it was the first time that the patients were hospitalized in Beijing Anding Hospital according to medical records; (b) the patients were at least 18 years old. The exclusion criteria included: (a) the patient was not of Chinese nationality; (b) the patient’s mental disorder diagnosis was not definitive. The raw dataset consists of individual-level data for each patient, including patient’s medical record number (a unique lifetime identification number), age, gender, admitting diagnosis, discharge diagnosis, the patient’s chief complaint, present medical history, past medical history, family history and personal history. 

### 2.2. Diagnoses of Mental Disorders

The patients were diagnosed by at least two mental health professionals (attending or senior doctors). Mental disorders are a set of mental and behavioral disorders as defined by the International Statistical Classification of Diseases and Related Health Problems (ICD-10). These disorders include organic, including symptomatic mental disorders (F00–F09), mental and behavioral disorders due to psychoactive substance use (F10–F19), schizophrenia, schizotypal and delusional disorders (F20–F29), mood (affective) disorders (F30–F39), neurotic, stress-related somatic disorders (F40–F48), behavioral syndromes associated with physiological disturbances and physical factors (F50–F59), disorders of adult personality and behavior (F60–F69), mental retardation (F70–F79), disorders of psychological development (F80–F89), behavioral and emotional disorders with onset usually occurring in childhood and adolescence (F90–F98) and unspecified mental disorder (F99). The inpatients were categorized according to their primary discharge diagnosis, which was more accurate than admitting diagnosis.

### 2.3. Suicidal Behaviors

According to the WHO report *Preventing suicide: A global imperative*, suicide refers to an act of deliberately killing oneself. Suicidal behaviors are defined as a range of behaviors including thinking about suicide (or suicide ideation), planning for suicide, attempted suicide (or incomplete suicide) and complete suicide [2]. For the purpose of this study, suicidal behaviors included “suicide ideation”, “planning for suicide”, and “attempted suicide”. 

Suicidal ideation concerns thoughts about or intent to suicide. Planning for suicide is defined as those with mental disorders who planned to die by suicide but had not attempted the act of suicide yet. Attempted suicide is defined as patients who have carried out suicidal behaviors but survived, saved by others or whose fatal actions were stopped. It refers to intentionally self-inflicted poisoning, injury or self-harm with a fatal intent. 

Suicidal behavior was one of the issues of most concern, which was routinely and mandatorily asked by professionals in Beijing Anding Hospital. Mental health specialists asked the patients and their relatives or guardians about the suicidal behaviors occurring 2 months before the patients admission to the hospital, and recorded whether or not the patients had suicidal behaviors. A new variable “suicidal behavior in past 2 months (0 = No, 1 = Yes)” was created according to these records.

### 2.4. Data Management and Analysis

We categorized the patients according to their primary discharge diagnosis, and manually divided them into groups for the convenience of our analysis. Inpatients’ suicidal behaviors were identified by researchers according to the information recorded in the “patient’s chief complaint”, “present medical history” and “past medical history”. Two professionals read all of these records independently and identified whether or not the inpatients presented suicide ideation or attempted suicide (as the important reason attend to the hospital) during the past 2 months right before being admitted in the hospital. A single identification for each inpatient would be made after combining two professionals' opinions (different opinions would be discussed and come to agreement). 

Data were managed and analyzed using SAS 9.4 (SAS Institute, Cary, NC, USA). We at first provided the frequency and percentage distribution of the inpatients with mental disorders by year and gender. Chi-square tests were used to compare the differences among inpatients with mental disorders by gender and year, and a linear-by-linear association was adopted to test the linear trends for each category of mental disorder. We graphed the frequency, trend and suicidal behavior rates for inpatients with mental disorders, frequency of inpatients with suicidal behaviors for each category of mental disorder, and the suicidal behavior rates of inpatients with each category of mental disorder by year. Any *p*-value less than or equal to 0.05 was considered statistically significant. 

## 3. Results

A total of 17,244 inpatients who were admitted to Beijing Anding hospital between 1 January 2010 and 31 December 2015, were included in our study. These inpatients were all Chinese citizens and no less than 18 years old. There were eight categories of inpatients with mental disorders, which were “mood disorders”, “schizophrenia, schizotypal and delusional disorders (psychotic disorders in short)”, “mental and behavioral disorders due to psychoactive substance use (substance use disorders in short)”, “organic, including symptomatic, mental disorders (organic mental disorders in short)”, “neurotic, stress-related and somatoform disorders (somatoform disorders in short)”, “mental retardation”, “behavioral and emotional disorders with onset usually occurring in childhood and adolescence (occurring in childhood and adolescence in short)” and “others”. About 53.2% of the total inpatients were mood disorders, followed by psychotic disorders, which accounted for 34.6%. Inpatients with mental disorders had increased in the 6-year period, especially in inpatients with mood disorders and schizophrenia, while the number of other mental disorders showed little change during 6 years. More details are shown in Table 1.

Table 2 presents the frequency and percentage of inpatients with mental disorders in Beijing Anding hospital by gender. Overall 52.6% of the inpatients with mental disorders were female. The proportions of mental disorders significantly differed between males and females. The proportion of female inpatients with mental disorders was larger than that of males (52.6% to 47.4%). The numbers increased steadily both in males and females, especially in inpatients with mood disorders and schizophrenia.

**Figure 1:**
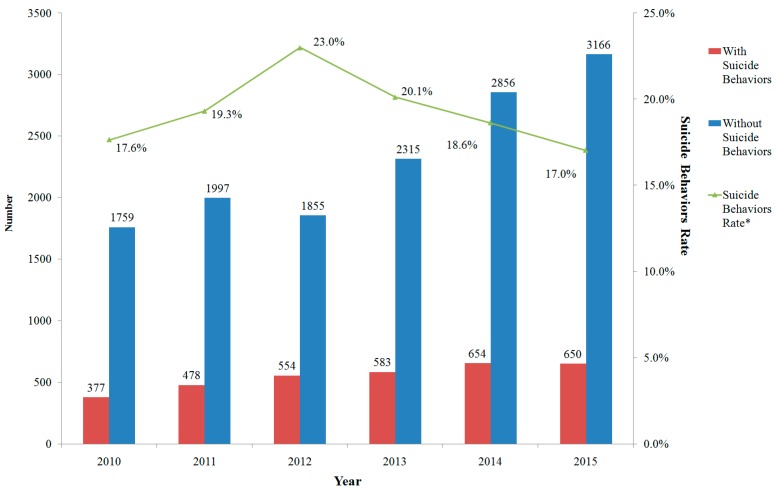
Numbers and suicidal behavior rates for inpatients with mental disorders in Beijing Anding Hospital, 2010–2015 (* *χ*^2^ = 39.6, *p* < 0.001; linear-by-linear association: *χ*^2^ = 4.4, *p* = 0.035).

Among the included subjects, 3296 of them were recognized as having suicidal behaviors. The rate of suicidal behaviors was 19.1%, and differed each year with a peak in 2012, as shown in Figure 1. The number of inpatients with suicidal behaviors increased steadily from 2010 to 2014, and slightly decreased in 2015. Suicidal behavior rates showed a significant linear trend during the 6 years studied.

**Figure 2:**
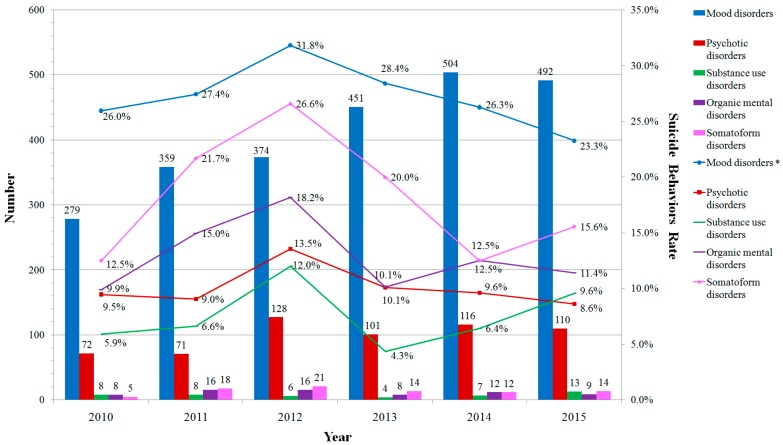
Numbers and suicidal behavior rates of inpatients with each category of mental disorders in Beijing Anding Hospital, 2010–2015 (* linear-by-linear association: *χ*^2^ = 8.4, *p* = 0.004).

Most of the inpatients with suicidal behaviors were those with mood disorders. The second group was inpatients with psychotic disorders, and the number increased greatly in 2012, then changed little from 2013 to 2015. The suicidal behavior rates in mood disorders were the highest among all inpatients, followed by “neurotic, stress-related and somatoform disorders”, “organic, including symptomatic mental disorders”, “schizophrenia, schizotypal and delusional disorders” and “mental and behavioral disorders due to psychoactive substance use”. Suicidal behavior rates among inpatients with mood disorders showed significant linear trend during 6 years. More details are shown in Figure 2.

**Figure 3:**
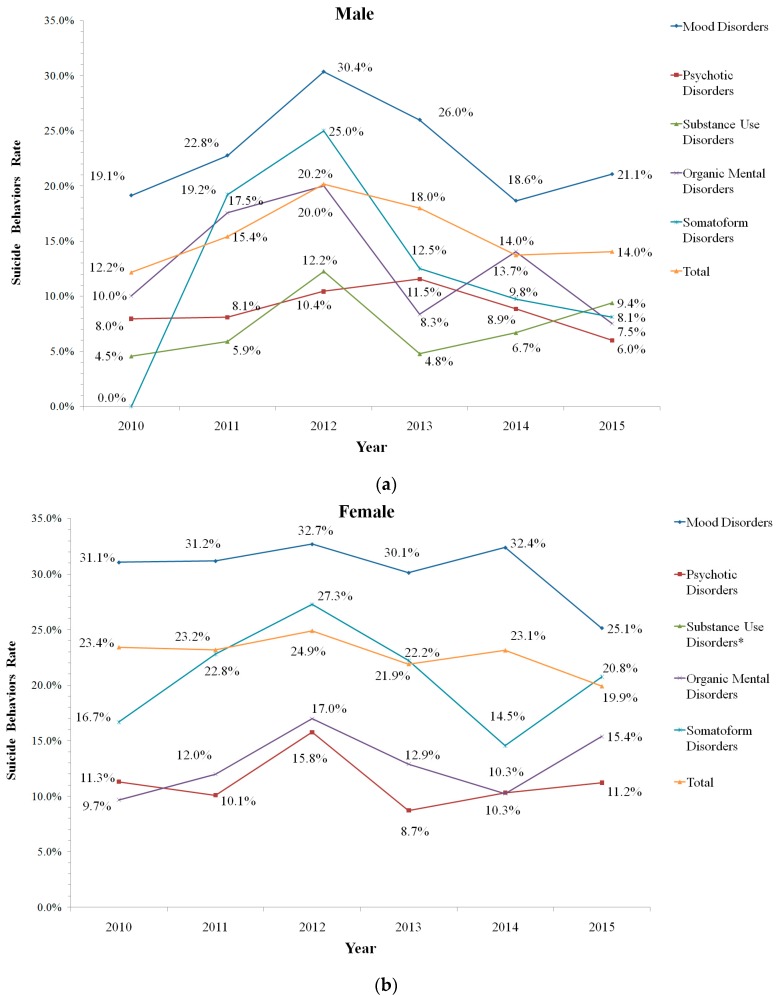
The suicidal behavior rates of (**a**) male and (**b**) female inpatients with mental disorders in Beijing Anding Hospital, 2010–2015. (* Substance Use Disorders was not shown because of the small numbers.).

The total suicidal behavior rate for the male inpatients was 15.3%. The suicidal behavior rates in mood disorders were the highest, followed by “neurotic, stress-related and somatoform disorders”, “organic, including symptomatic mental disorders”, “schizophrenia, schizotypal and delusional disorders” and “mental and behavioral disorders due to psychoactive substance use” (Figure 3a). In female inpatients, the suicidal behavior rate was 22.6%. The female suicidal behavior rates for mood disorders were much higher than those of males, and it was also the highest overall. 

Female inpatients with “neurotic, stress-related and somatoform disorders” ranked in second place in terms of suicidal behavior rates. The following were “organic, including symptomatic mental disorders”, “schizophrenia, schizotypal and delusional disorders” (Figure 3b). More details are shown in Figure 3.

The rates of suicidal ideation and attempted suicide differed in the different mental disorder categories. The inpatients with mood disorders had the highest rates of suicide ideation. The following were inpatients with somatoform disorders, psychotic disorders, organic mental disorders, psychotic disorders, substance use disorders and mental retardation. The rates of attempted suicide followed the same order. Among inpatients with mood disorders, the rate of suicidal ideation was significantly larger than the rate of attempted suicide. While among inpatients with other mental disorders, there were no significant differences. More details are shown in Table 3.

## 4. Discussion

Few studies have investigated the tendency and suicidal behaviors among Chinese adult inpatients with different types of mental disorders, especially studies with large sample sizes. In this study, we included 17244 inpatients and found that the number of inpatients with mental disorders increased from 2010 to 2015, and the number of inpatients with suicidal behaviors also increased, especially in inpatients with mood disorders and schizophrenia.

The prevalence of mental disorders was 17.5% in China, one of the highest rates in the world [14], which makes it a serious public health issue. However, mental health had not received sufficient attention in China for various historical reasons, and the government budget for mental health care was very low compared to the extent of burden of mental disorders [15]. 

According to our data, the inpatients with mental disorders increased steadily from 2010 to 2015 (except for 2012). There were a few possible interpretations of this finding. First, the number of psychiatric patients in China increased these years. A second interpretation was that the awareness from the public on the mental health improved especially after the enforcement of the Mental Health Law of the People’s Republic of China since 2013, and an increasing number of people with mental illness sought help at psychiatric hospitals. 

In this study, inpatients with mood disorders and psychotic disorders represented the overwhelming majority among all psychiatric inpatients, accounting for 53.2% and 34.6%, respectively. This was followed by the substance use disorders (3.7%), organic mental disorders (3.1%) and somatoform disorders (2.7%). It was different for a previous study in China, which included the natural population and reported people with substance use disorders were much more than those with psychotic disorders [14]. The different results might be attributed to the different populations adopted in the two studies.

Mental disorders show a clear sex distinction across the world [16]. Although findings from our study found that both the male and female inpatients with mood disorders took the majority among all categories of mental disorders, the proportions of male inpatients were much lower than that of females (the difference was about 10% each year). A similar situation happened among inpatients with somatoform disorders (male inpatients also represented a lower percentage). There was evidence that women reported a higher number of physical and psychological symptoms than men, and comorbidity was also more common among women than men, and it often took the form of a co-occurrence of somatoform disorders [16].

Almost all the studies have shown that substance use disorders are much more common among men than women [2,3,17]. Our results also indicated that there were much more male inpatients with substance use disorders than female ones. The individuals with substance use disorders in the West (North America and Europe) were much more than that in China especially for the people with alcohol abuse [2]. The reason might be that alcohol abuse was considered to be a habit rather than a mental disorder in Chinese culture, and few people suffering from alcohol abuse went to hospital for help. Thus, the issue of substance use disorders might be far underestimated in China.

In contrast to mood disorders and substance use disorders, schizophrenia and other mental disorders did not show clear differences in proportion between male and female inpatients in our study, which was consistent with former studies [14,18]. However, Phillips et al. [19] found that relative risk of suicide in rural residents with schizophrenia comparing those without was higher in men than in women, but in urban areas, the ratio was lower in men than women. Further studies are needed concerning the underlining mechanism.

Mental disorders are one of the most important factors investigated in suicides. In the West, over 90% of suicides are associated with mental illness [12,20], while in China the proportion varied in different studies [2]. In a nationally representative psychological autopsy study, Phillips et al. [13] reported that 40% of suicide victims were diagnosed with depression, 7% with schizophrenia, and 7% with alcohol dependence. Zhang et al. [21] found that 76% of rural suicide victims had a diagnosable mental illness in a psychological autopsy study. There were few studies concerned on suicide rate among confirmed patients with mental disorders in China. The suicide rates of each category of mental disorder and the relative risks of suicide among different mental disorders is still a mystery.

Our study included ideation in suicidal behaviors. WHO report also included ideation in suicidal behavior for the purpose of simplicity since suicide ideation might more likely lead to suicide actions as the course of mental disorders last for years, though there is meaningful ongoing academic dialogue about such complex issue [2]. We intended not to include deaths from suicide in this study, and in fact there were no such cases in this hospital during these 6 years.

The overall suicide rates in China decreased sharply in the past two decades. The rates were about 23 per 100,000 people [3] in 1990 and about 8 per 100,000 people in 2006. From 2006 to 2011, the suicide rates showed a fluctuation of around 8 per 100,000 [22]. The exact number of suicide rate in China was still controversial because different sources of data were used in previous studies, but a sharp reduction of the suicide rate from the 1990s to the 2010s is generally recognized. The reason is still unclear. According to some points of view, the reduction of pesticide toxicity might play an important role. Meanwhile, the rural labor migration from rural to urban areas might also be an important reason. Our study showed that the numbers of inpatients with suicidal behaviors increased from 2010 to 2015, yet the suicidal behavior rates showed a trend of fluctuations around 17%. The suicidal behavior rates were also fluctuating during the 6 years and showed a reduction tendency according to linear-by-linear association analysis.

Previous studies suggested that a large number of individuals with schizophrenia attempt suicide during the course of their illness [16]. Radomsky et al. [23] reported that 30% of patients with schizophrenia had attempted suicide at least once during their lifetime, while Caldwell’s study found that about 10% of persons with schizophrenia died by suicide [24]. However, the suicide rate in Chinese people with schizophrenia was lower than that in the West. Phillips et al. [19] found that the suicide rate in individuals with schizophrenia aged 15 years and older was 6.8 per 1000 people per year. The results of our study found that the suicidal behavior rate of schizophrenia was around 10%. The suicidal behavior rates of inpatients with schizophrenia were relatively low both in male and female inpatients compared with the West [25,26]. Future studies should be carried out to find the mechanism or cause regarding this phenomenon. 

Alcohol dependence, one of the most important substance use disorders, might lead to more suicidal behaviors than mood disorders and schizophrenia in the West [2,27]. However, the suicidal behavior rates of inpatients with substance use disorders were the lowest in male inpatients in this study (few female patients with substance use disorders). As stated above, substance use disorders, especially the alcohol dependence (over 90% among patients with substance use disorders, data was not shown) might potentially be a much more serious problem in China, which called for more studies.

The suicidal behavior rate abruptly rose in 2012 and then declined, which seemed abnormal, because there was a reconstruction for one inpatient area in 2012 in Beijing Anding hospital (all together 15 inpatient areas), and a number of patients without suicidal behaviors (relatively less dangerous) in 2012 might not be admitted in the hospital. 

Nock et al. [28] conducted survival models to examine the associations between individual disorders and subsequent suicidal behaviors using the National Comorbidity Survey data. Mood disorders played a major role in accounting for the onset of suicide ideation, followed by anxiety disorders, impulse-control disorder and substance use disorder. Besides, predictive effects of mood disorders to suicidal ideation were larger than that to attempted suicide. In our study, we also found that mood disorders and neurotic, stress-related somatoform disorders were the top two most important mental disorders associated with suicidal behaviors, and among inpatients with mood disorders, the rate of suicidal ideation was larger than that of attempted suicide. 

In this study our outcome measure represents suicidal behavior in the time recently before admission, i.e., it represent if they present at hospital with previous suicidal behavior, thus from our data we cannot comment on suicidal behavior in inpatients while admitted to hospital. We would conduct further studies to figure out which diagnostic categories displayed more suicidal behaviors during an inpatient stay, which would be better to apply for tailor made preventive efforts for an inpatient stay. Another limitation for this study was that it could only provide clues for the trend of inpatients with mental disorders in China. The data were limited by the admission rate, which might influence the results, and the Berkson’s selection bias could not be eliminated. As was mentioned above, combining medical records from two or more hospitals was not possible in China. The sample size in this study was relatively small comparing to some western studies, which might lead to controversial inferences, especially for subgroups with small sample size. However, researches on mental disorders and suicidal behaviors were still in development stage in China, and there is a scarcity of studies about mental patients using large sample size survey. This study was a beneficial practice to provide ideas and a basis for further studies.

## 5. Conclusions

The number of inpatients increased steadily during recent years, and mood disorders and schizophrenia were the top two serious categories of mental disorders. The presence of suicidal behaviors varied in people with different types of mental disorders. Mood disorders might be the most important category of mental disorders associated with suicidal behaviors. Identification of specific suicidal behaviors risk for each type of mental illness would help tailor prevention efforts.

## Figures and Tables

**Table 1:** The frequency and percentage of inpatients with mental disorders in Beijing Anding Hospital, 2010–2015 (%).

Mental Disorders	2010	2011	2012	2013	2014	2015	Total
Mood disorders	1075 (50.3)	1308 (52.8)	1175 (48.7)	1587 (54.8)	1918 (54.6)	2114 (55.4)	9177 (53.2)
Psychotic Disorders	760 (35.6)	785 (31.7)	945 (39.2)	1001 (34.5)	1206 (34.3)	1276 (33.4)	5973 (34.6)
Substance use Disorders	136 (6.4)	121 (4.9)	50 (2.1)	92 (3.2)	109 (3.1)	136 (3.6)	644 (3.7)
Organic mental disorders	81 (3.8)	107 (4.3)	88 (3.7)	79 (2.7)	96 (2.7)	79 (2.1)	530 (3.1)
Somatoform disorders	40 (1.9)	83 (3.4)	79 (3.3)	70 (2.4)	96 (2.7)	90 (2.4)	458 (2.7)
Mental retardation	16 (0.7)	39 (1.6)	39 (1.6)	33 (1.1)	45 (1.3)	54 (1.4)	226 (1.3)
Occurring in childhood and adolescence	18 (0.8)	24 (1.0)	24 (1.0)	28 (1.0)	32 (.9)	62 (1.6)	188 (1.1)
Others	10 (0.5)	8 (0.3)	9 (0.4)	8 (0.3)	8 (0.2)	5 (0.1)	48 (0.3)
Total	2136 (12.4)	2475 (14.4)	2409 (14.0)	2898 (16.8)	3510 (20.4)	3816 (22.1)	17,244 (100)

**Table 2:** The numbers of inpatients with mental disorders in Beijing Anding Hospital by gender, 2010–2015 (%).

Mental Disorders	2010		2011		2012		2013		2014		2015		Total	
	Male	Female	Male	Female	Male	Female	Male	Female	Male	Female	Male	Female	Male	Female
Mood disorders	460 (42.0)	615 (59.0)	580 (47.0)	728 (58.6)	438 (45.3)	737 (51.1)	654 (49.4)	933 (59.2)	853 (50.7)	1065 (58.3)	964 (51.5)	1150 (59.2)	3949 (48.3)	5228 (57.6)
Psychotic Disorders	415 (37.9)	345 (33.1)	408 (33.1)	377 (30.4)	393 (40.6)	552 (38.3)	485 (36.7)	516 (32.8)	576 (34.2)	630 (34.5)	634 (33.8)	642 (33.0)	2911 (35.6)	3062 (33.8)
Substance use disorders	132 (12.1)	4 (0.4)	119 (9.7)	2 (0.2)	49 (5.1)	1 (0.1)	84 (6.3)	8 (0.5)	105 (6.2)	4 (0.2)	128 (6.8)	8 (0.4)	617 (7.6)	27 (0.3)
Organic mental disorders	50 (4.6)	31 (3.0)	57 (4.6)	50 (4.0)	35 (3.6)	53 (3.7)	48 (3.6)	31 (2.0)	57 (3.4)	39 (2.1)	40 (2.1)	39 (2.0)	287 (3.5)	243 (2.7)
Somatoform disorders	10 (0.9)	30 (2.9)	26 (2.1)	57 (4.6)	24 (2.5)	55 (3.8)	16 (1.2)	54 (3.4)	41 (2.4)	55 (3.0)	37 (2.0)	53 (2.7)	154 (1.9)	304 (3.4)
Mental retardation	12 (1.1)	4 (.4)	26 (2.1)	13 (1.0)	20 (2.1)	19 (1.3)	21 (1.6)	12 (0.8)	27 (1.6)	18 (1.0)	33 (1.8)	21 (1.1)	139 (1.7)	87 (1.0)
Occurring in childhood and adolescence	12 (1.1)	6 (0.6)	13 (1.1)	11 (0.9)	5 (0.5)	19 (1.3)	10 (0.8)	18 (1.1)	18 (1.1)	14 (0.8)	34 (1.8)	28 (1.4)	92 (1.1)	96 (1.1)
Others	3 (0.3)	7 (0.7)	4 (0.3)	4 (0.3)	3 (0.3)	6 (0.4)	5 (0.4)	3 (0.2)	5 (0.3)	3 (0.2)	3 (0.2)	2 (0.1)	23 (0.3)	25 (0.3)
Total *	1094 (51.2)	1042 (48.8)	1233 (49.8)	1242 (50.2)	967 (40.1)	1442 (59.9)	1323 (45.7)	1575 (54.3)	1682 (47.9)	1828 (52.1)	1873 (49.1)	1943 (50.9)	8172 (47.4)	9072 (52.6)

* Difference by year, *χ*^2^ = 77.5, *p* < 0.001.

**Table 3:** The numbers and rates of suicidal ideation and attempted suicide for each category of mental disorders.

Mental Disorders	Suicidal Ideation		Attempted Suicide	
	Number	Rate (95% CI) %	Number	Rate (95% CI) %
Mood disorders	1360	14.8 (14.1, 15.5)	1099	12.0 (11.3, 12.6)
Psychotic Disorders	276	4.6 (4.1, 5.2)	322	5.4 (4.8, 6.0)
Substance use disorders	25	3.9 (2.4, 5.4)	21	3.3 (1.9, 4.6)
Organic mental disorders	28	5.3 (3.4, 7.2)	41	7.7 (5.5, 10.0)
Somatoform disorders	46	10.0 (7.3, 12.8)	38	8.3 (5.8, 10.8)
Mental retardation	8	3.5 (1.1, 5.9)	6	2.7 (0.6, 4.8)
Others	13	16.2 (4.1, 28.3)	13	14.7 (3.2, 26.2)
Total	1756	10.2 (9.7, 10.6)	1540	8.9 (8.5, 9.4)
